# A Professional Development Model for Training Biology GTAs in Equitable Teaching: Implementation and Evaluation at Two Minority-Serving Institutions

**DOI:** 10.1093/iob/obag052

**Published:** 2026-09-11

**Authors:** J R Vidrio, O M Takano, L M Hartley, J C Danek, C P Hedberg, S Kuchynka, P T Le, S Levine, M C Murphy, J R Pollard, K Ryan, F F Scheideman, S D Spong, S Maliakal-Witt

**Affiliations:** Department of Integrative Biology, University of Colorado Denver, 1151 Arapahoe St, Denver CO 80204, USA; Coalition of Urban Serving Universities, 1220 L St NW Suite 1000, WA, DC 20005, USA; Department of Biology, University of New Mexico, Castetter Hall MSC03-2020, 219 Yale Blvd NE, Albuquerque, NM 87131-0001, USA; Department of Integrative Biology, University of Colorado Denver, 1151 Arapahoe St, Denver CO 80204, USA; Coalition of Urban Serving Universities, 1220 L St NW Suite 1000, WA, DC 20005, USA; Department of Biology, University of New Mexico, Castetter Hall MSC03-2020, 219 Yale Blvd NE, Albuquerque, NM 87131-0001, USA; Equity Accelerator, 1101 E 10th St Bloomington, IN 47405, USA; Department of Integrative Biology, University of Colorado Denver, 1151 Arapahoe St, Denver CO 80204, USA; Center for Teaching and Learning, University of New Mexico, MSC05 3020 1 University of New Mexico, Albuquerque, NM 87131-0001, USA; Association of Public Land-Grant Universities, 1220 L St NW Suite 1000, WA, DC 20005, USA; Equity Accelerator, 1101 E 10th St Bloomington, IN 47405, USA; Center for Teaching and Learning, University of New Mexico, MSC05 3020 1 University of New Mexico, Albuquerque, NM 87131-0001, USA; Equity Accelerator, 1101 E 10th St Bloomington, IN 47405, USA; Department of Integrative Biology, University of Colorado Denver, 1151 Arapahoe St, Denver CO 80204, USA; Center for Teaching and Learning, University of New Mexico, MSC05 3020 1 University of New Mexico, Albuquerque, NM 87131-0001, USA; Department of Biology, University of New Mexico, Castetter Hall MSC03-2020, 219 Yale Blvd NE, Albuquerque, NM 87131-0001, USA

## Abstract

Graduate Teaching Assistants (GTAs) play a critical role in undergraduate biology education, yet rarely receive training in the inclusive teaching practices that support student belonging, growth mindset, and persistence in STEM. Many GTAs are future faculty, department chairs, and course designers, and investing in their development early represents an opportunity to improve undergraduate experiences at scale. Here we describe the SEP-GTA professional development model, adapted from the Student Experience Project (SEP) for university faculty, and its implementation at two minority-serving research universities. The model is founded around three pillars: a GTA community of practice with peers, evidence-based inclusive teaching practices rooted in growth mindset and belonging research, and collection of formative feedback from students. We present program evaluation data showing that after participating in the program, GTAs had higher endorsement of growth mindset beliefs (ranging from +5% to +11% across items), greater familiarity (+16%) and comfort with (+12%) using evidence-based teaching practices, increased interest in professional development (+18%), and greater perception of departmental support (+11%). After the training, 13 of the 16 specific teaching strategies were used by more than 80% of GTAs in their introductory biology laboratory and lecture courses. The SEP-GTA resources are freely available and flexible enough to be adapted across institutional contexts, making this model a practical starting point for institutions looking to invest in GTA professional development and build learning environments where all students feel they belong and can succeed.

## Introduction

### The impact of GTAs in undergraduate biology

Graduate Teaching Assistants (GTAs) play a key role in undergraduate biology education, yet they rarely receive training in creating a classroom that fosters a growth mindset and a sense of belonging, which are critical for undergraduate persistence (Walton and Cohen 2011; Canning et al. 2021). Sundberg et al. (2005) found that GTAs provided the majority of instruction for laboratory courses at comprehensive (71%) and research universities (91%). According to the American Association of University Professors Department of Research and Policy Report, GTA employment increased by 44% from 2002 to 2021, while full-time and part-time faculty positions have declined by 19% (Colby 2023). GTAs are often instructors of introductory biology laboratory courses, which typically enroll many first-year students. There is evidence that experiences and grades in the first year of university are important for persistence in undergraduate degree programs (Kuh et al. 2008; Stewart et al. 2015; Jenkins and Bailey 2017). Given the pivotal role that GTAs can play in undergraduate education, we suggest they should receive specialized training to support undergraduates' experiences in the classroom.

### Gaps in GTA professional development

Despite GTAs’ importance in undergraduate education, professional development training for GTAs has historically been inconsistent or non-existent (Boyer Commission on Education Undergraduates in the Research University 1998; Prieto and Meyers 1999; Golde and Dore 2001; DeChenne et al. 2012). Furthermore, Prieto and Myers (2001) found that 25% of GTAs had no supervision while teaching. GTA training often focuses on activities such as classroom management rather than pedagogical practices that improve student learning and persistence (Luft et al. 2004; Gorsuch 2012). With a focus on biology, Schussler et al. (2015) found that formal pedagogical training for teaching assistants is not offered at many institutions, which is similar to findings for STEM GTAs more broadly (Connolly et al. 2016). Inclusive pedagogy is increasingly recognized as an important focus of GTA professional development (e.g., Agresar et al. 2019; Fong and Ciston 2020; Shortlidge 2022; Batterton et al. 2024; Goodwin et al. 2025). The work we present here illustrates that training that focuses on promoting growth mindset and belonging in undergraduate students represents a promising lever for GTAs to increase their use of evidenced-based inclusive teaching practices, which have been shown to improve undergraduate student experiences and close equity gaps in STEM (Walton and Cohen 2011; Canning et al. 2021). In introductory biology specifically, inclusive and active pedagogies have improved grades and narrowed ethnic achievement gaps compared to traditional lecture-based interventions (Dewsbury et al. 2022).

### GTAs as future faculty

GTAs teaching undergraduates in biology are not just temporary instructors—many are future faculty mentors, department chairs, or course designers. Research suggests that many GTAs actively want teaching to be central in their careers (Golde and Dore 2001; Tanner and Allen 2006) and those who develop strong professional teaching identities early become more competitive faculty candidates (Sauermann and Roach 2012; Kendall et al. 2013). Research further shows that greater engagement in teaching development during doctoral training is associated with higher teaching self-efficacy and increased likelihood of securing faculty positions (Connolly et al. 2016). Investing in GTA training in evidence-based belonging and growth mindset practices, therefore, represents an opportunity to improve undergraduate experiences at scale.

## The SEP-GTA professional development model

Here we present a GTA professional development (PD) model that goes beyond one-off training workshops by embedding evidence-based inclusive teaching practices within a sustained community of practice where GTAs learn and grow alongside their peers. The GTA PD model described in this manuscript is based largely on the Student Experience Project (SEP), a national initiative that has engaged nearly 300 college instructors to implement equity-focused teaching practices (Student Experience Project 2025a). SEP data from the 2020–2021 academic year showed that the percentage of students with positive experiences of belonging, identity safety, and trust in the instructor increased by ∼10.5% each semester in SEP courses. The SEP supported instructors in implementing inclusive practices such as using growth mindset statements in their written and verbal communications with students, ensuring that course policies were equitable to students of all identities, and encouraging student connections through activities like ice-breakers and group work. As students’ experiences improved, they were significantly more likely to earn a grade of A or B, and less likely to withdraw or earn a D or F in the course where faculty implemented equity focused practices. Students from structurally disadvantaged backgrounds experienced the largest benefit, including women, students from racial/ethnic backgrounds under-represented in STEM disciplines, first-generation students, and students experiencing financial stress (Student Experience Project 2022). Our motivations for adapting SEP resources for GTAs include: SEP resources have been shown to be adaptable and successful in a wide variety of contexts, and we believed they would be tractable for GTAs. The majority of undergraduates at research institutions in the US will receive instruction from GTAs, suggesting that GTA training could have a large impact on student success. In this current work, we adapted the SEP resources to be more applicable for the GTA context. Here we describe the SEP-GTA program, its implementation at two minority-serving institutions (MSI), and present evidence of the program’s impact on GTA mindset beliefs, teaching self-efficacy, and use of evidence-based teaching practices.

### Program overview

The SEP-GTA PD model is organized around three pillars (Fig. 1): a community of practice through which GTAs learn and grow alongside peers, learning and implementation of evidence-based inclusive teaching practices, and collection of and reflection on formative feedback from students. Each pillar is described in detail below with the goal that others may apply this model to guide GTA PD within their own institutional contexts. The SEP-GTA resources are freely available through the Student Experience Project (2025b).

**Fig. 1:**
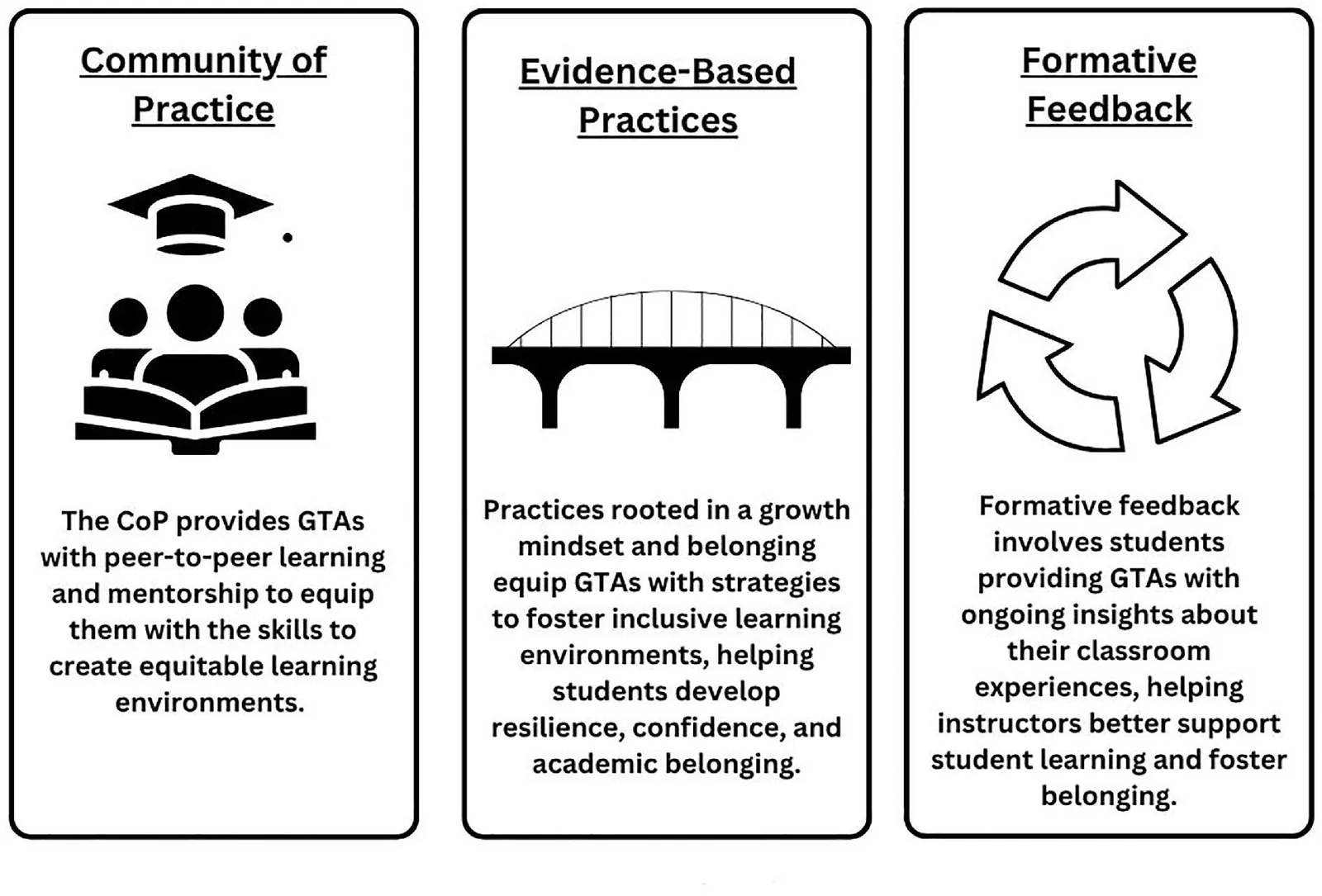
The Student Experience Project PD model, adapted for use by GTAs, includes a community of practice for GTAs, learning and implementation of specific evidence-based inclusive teaching practices, and reflecting on formative feedback from students about their experiences.

### Pillar 1—A community of practice

A community of practice (CoP) brings people together around a shared goal; in this case, for GTAs to create classroom environments where all students can thrive. The CoP approach allows participants to learn with and from one another over time rather than through a single training event (Lave and Wenger 2001; Wenger et al. 2002). This matters because research shows that one-time workshops are largely ineffective at changing instructional practices, while sustained, collaborative learning experiences that include implementation, reflection, and peer feedback produce meaningful shifts in how instructors teach (Darling-Hammond et al. 2017). In STEM contexts specifically, instructors who participated in a CoP were significantly more likely to use student-centered, active learning practices compared to those who did not, which increased student engagement (Tomkin et al. 2019). In biology specifically, instructors participating in small peer groups reported that the group structure kept them engaged over time; without it, several noted they might never have discussed their teaching with colleagues at all (McCourt et al. 2017).

In our program, the CoP gives GTAs a space to learn a new teaching practice, try it in their classroom, and then come back to debrief with peers who were having similar experiences. This cycle of learn, implement, and reflect, repeated throughout the semester, is what distinguishes this program from many professional development trainings. In a CoP, GTA participants are able to workshop teaching materials together and collectively solve problems, build connections with their peers throughout the semester, and share knowledge across biology courses.

### Pillar 2–Evidence—based practices

The SEP-GTA program draws on two well-established bodies of research in social psychology: growth mindset and belonging. These constructs informed the specific classroom practices GTAs learned and implemented throughout the program.

#### Growth mindset

Growth mindset is the belief that ability is not fixed but can be developed over time through effort, feedback, and effective strategies (Dweck 2006). Research shows that a growth mindset positively influences academic outcomes including grades, learning, and persistence (Rattan et al. 2018). Importantly, encouraging students to adopt a growth mindset is insufficient if the learning environment does not also provide authentic opportunities for growth through feedback and effective strategies (Muenks et al. 2020; Walton and Yeager 2020). Fixed mindset beliefs communicated through classroom policies, practices, and instructor interactions can engender identity threat (the worry about being viewed negatively on the basis of social group stereotypes), particularly for students from historically underserved groups in STEM (Canning et al. 2019, 2021; Murphy and Reeves 2019). Classroom cultures that support growth mindset ameliorate identity threat and benefit all students, with the greatest gains for students from historically underserved groups (Emerson and Murphy 2015; Murphy and Reeves 2019).

#### Belonging

A sense of belonging, feeling accepted, valued, and connected within a community, is fundamental to student persistence in higher education (Strayhorn 2012). Students who feel they do not belong are more likely to have lower academic outcomes and leave their major or institution altogether (Tinto 1993; Walton and Cohen 2007). While belonging uncertainty is a normal part of the college experience, it is more commonly experienced by students from historically underserved groups (Hurtado and Carter 1997; Stephens et al. 2012; Strayhorn 2012). Social-psychological interventions that address belonging uncertainty have been shown to increase engagement, help-seeking, and persistence, particularly for students from underserved groups (Walton et al. 2015; Walton and Cohen 2011; Brady et al. 2020). Related to belonging is the concept of identity safety, the degree to which students feel their social identities are valued and respected in the learning environment (Murphy and Taylor 2012), which the SEP practices are specifically designed to foster.

Research on belonging and growth mindset informed a set of practice guides developed by Equity Accelerator (available at https://accelerateequity.org/resource/practices-library/) that GTAs used throughout the program. Practice guides introduce instructors to the evidence base behind a specific teaching strategy, provide examples of what these strategies look like in the classroom, and offer guidance on common pitfalls to avoid. For example, the Belonging Stories practice guide helps GTAs craft and share a story about their own experiences with belonging uncertainty, with the goal of normalizing challenges for students, and communicating that belonging can improve over time.

### Pillar 3—Formative feedback

GTAs typically receive course feedback from students at the end of the semester when they are no longer able to act upon it, and often on matters they have little to no control over, such as existing course policies. Our model addresses this issue directly by having GTAs collect short anonymous feedback (e.g., surveys, exit tickets) from their students throughout the semester, focusing specifically on areas within the GTA’s control and on student experiences related to mindset and belonging in the classroom (Mitra 2018; Conner et al. 2024). Throughout this paper, we define formative feedback as feedback given to GTAs by their undergraduate students, which can provide GTA instructors with insights into student experiences while modeling growth mindset principles and demonstrating that student perspectives are genuinely valued.

Identifying strengths and areas for improvement is a process that is particularly valuable for normalizing the challenges of being a new instructor and generating shared solutions (Camarao and Din 2023). Based on student feedback, GTAs select an evidence-based inclusive practice to implement and later reassess whether student experiences have shifted. Collecting and responding to feedback regularly during the semester rather than a one-time measurement allows for real-time improvements. For undergraduate students, this process signals that their instructor is genuinely invested in their experience and that their feedback will have a real impact on their course (Steele and Aronson 1995; Walton and Cohen 2011).

### Partnership and institutional context

The partners involved in this project previously worked together as part of the SEP in 2020 (Student Experience Project 2022). The team included four partners: two national organizations with a focus on higher education and biology departments at two universities. The “organizational partners” are from leading national organizations and have expertise in social psychology, institutional change, teaching and learning, and evidence-based inclusive practices in STEM higher education. The “university partners” were the Rocky Mountain Institution, a research university located in the Mountain West, and the Southwestern Institution, a research university located in the Southwest. Both institutions are Hispanic Serving Institutions, and the Rocky Mountain Institution is also designated as an Asian American Native American Pacific Islander Serving Institution. The Rocky Mountain Institution has ∼40% underrepresented students and the Southwestern Institution has ∼60% underrepresented students. This partnership provided the collective expertise to adapt the SEP practices for GTAs.

The universities engaged multiple stakeholders at their locations, which included two Lead GTAs per university, faculty supervisors of GTAs, and individuals with expertise and experience using evidence-based teaching practices. The GTA participants in this study taught primarily laboratory courses ranging from Introductory Biology for non-majors and majors to upper-division specialty courses (e.g., Botany, General Vertebrate Zoology; see Supplemental Table S3 for a full list of courses taught by GTAs at each Institution). As is typical of research universities, the majority of laboratory courses in biology at these institutions are taught by GTAs. Leaders at each university, including department chairs and upper administrators, were committed to piloting the new SEP-GTA training model.

### Adapting the SEP materials for GTAs

Unlike faculty, GTAs occupy a distinct role as both students and instructors, where their levels of autonomy vary across institutions, departments, and even within individual courses. In adapting the faculty SEP materials for GTAs, our team identified four key aspects of the GTA context. GTAs typically have less autonomy in developing and delivering classroom curriculum than faculty (Winters and Matusovich 2011; Flaherty et al. 2017; Freeman et al. 2025a), are unlikely to have prior teaching experience (Gardner and Jones 2011), and have time constraints (Cho et al. 2011). Additionally, GTAs are often closer to their students than faculty in terms of their career stage and life stage, which presents both opportunities and challenges related to power dynamics (Westwood and Srivastava 2025).

Three months before implementation of the programs, all partners met to review and discuss SEP resource guides for the evidence-based teaching practices (Student Experience Project 2025a). The Lead GTAs were involved as full collaborators in this review. Based on the review, we grouped the practices into three categories: essential, expansion, and collaborative (Table 1). Essential practices were those the Lead GTAs found most relevant and easy to implement in the GTA context. Expansion practices were practices that GTAs could use to deepen their engagement, but might need more time for consideration and implementation. Collaborative practices were those targeting elements like syllabus language and course policies that GTAs typically do not have autonomy to change without collaborating with their teaching supervisor.

**Table 1:** Student experience project classroom practices for GTAs.

Category	Practice	Description
**Essential:** Practices that GTAs found were most relevant to their roles and highly impactful for student success.	Establishing expectations	GTAs can establish growth mindset culture in the classroom by establishing course expectations in the first week of class.
	Wise feedback framing statements	Wise feedback pairs critical feedback with a statement of high standards and assurance that the student is capable of meeting them, which can help GTAs deliver feedback to students in ways that engender trust and growth.
**Expansion:** Practices for GTAs to deepen their engagement but may require additional time or consideration for implementation.	Belonging stories	GTAs can author and share a belonging story to normalize challenges in college.
	Encouraging connections	Suggestions for cultivating relationships between GTAs and their students, and among learners, to facilitate a more socially connected learning environment.
**Collaborative:** Practices that GTAs and faculty can discuss and implement together to create cohesion across the instructional team.	Syllabus review	A step-by-step guide for using syllabus development or revision as a tool for creating a course that promotes equity, belonging, and growth for all students.
	Policy review	A step-by-step guide for crafting course policies that promote equity and acknowledge and accommodate the lived experiences of diverse student populations.

All practice guides are available on the SEP website https://studentexperienceproject.org/gta/practices/.

Stakeholder engagement was a crucial component to GTAs being able to implement practices in the classroom. Prior work has shown that practitioners who face resistance to new teaching approaches, such as a negative course evaluation, need institutional support that protects and rewards continued use of those practices, or they may revert to their original approach (Dewsbury et al. 2022). At both universities, department chairs and deans provided institutional support for the program, while GTA supervisors and lab coordinators were engaged operationally to ensure GTAs felt supported in implementing practices in their classrooms. This alignment is especially critical when GTAs are not instructors of record; if a GTA is implementing growth mindset practices in a lab while the faculty instructor of record signals fixed mindset beliefs in the main course, those efforts can be directly undermined.

### Program implementation

Here we outline implementation at each participating university. Implementation included formation of GTA communities of practice; GTA learning and implementation of evidence-based practices in their classrooms; and collection, reflection, and response to formative feedback from their students by GTAs. Both campuses involved the Lead GTAs in designing and delivering a series of CoP sessions. Recruitment for GTA training was open to all GTAs in the department at both sites. Both universities created a plan for five CoP sessions that centered around SEP-GTA practice guides (Student Experience Project 2025a). The general structure of each session included (1) exposure to the topic through readings or videos, (2) exploration of the topic through activities (e.g., group discussion, individual reflective writing, practice/role-play, guest speaker with Q&A), and (3) time for the GTA to plan how they would implement the practice in their course and get feedback from their students. The CoP sessions often included some context specific to the university (e.g., disaggregated student enrollment data for the Department of Biology to illustrate diversity of students) and activities specific to the GTAs (e.g., practicing Wise Feedback using assignments from the course they were teaching). See Supplement Table S1 for a skeleton guide of sessions with examples from implementation at the two universities.

One university embedded the workshops into an existing pedagogy course required for GTAs, while the other created a new workshop series in coordination with experts from their Center for Teaching and Learning. Participation in the novel workshop series was incentivized with a stipend for program completion at both universities. The CoP at both universities included five in-person meeting times with asynchronous work (e.g., readings, reflective writing, gathering student feedback) in between meetings. Teams at both universities included very similar content in their workshops and targeted essential, expansion, and collaborative practices (Table 1). At both universities, GTAs collected regular feedback from their students using low-effort tasks for students, such as exit tickets (brief end of class check-ins). Student feedback was discussed in the session times and/or during the weekly GTA lab preparatory meeting, which was separate from the CoP sessions. GTAs at one of the universities submitted reflections as part of their course assignment and the instructor gave them feedback. GTAs at the other university kept a portfolio for each topic that included their reflections on feedback.

## Program evaluation

We conducted a program evaluation to assess the impact of the SEP-GTA PD model on participating GTAs. To do so, we surveyed GTAs at the beginning and end of the program to see if participation was associated with changes in GTA beliefs and perceptions about inclusive teaching, and to determine which evidence-based teaching practices GTAs reported using in their classrooms.

### IRB information

This program evaluation was reviewed and approved by the Institutional Review Board Office at the Rocky Mountain Institution and the Southwestern Institution. The program evaluation was determined exempt and assigned protocol #23-0892 at the Rocky Mountain Institution. At Southwestern Institution, it was classified as minimal risk and received an expedited review and was assigned protocol #2303046372. No individual who could determine a GTA’s grade saw individual-level responses to the survey and only saw responses after the conclusion of the program.

### Participants

At both institutions, we advertised the opportunity to all GTAs (MS and PhD students who had varying levels of teaching experience) who taught in the departments of biology. All GTAs who applied were accepted into the community of practice. All GTAs taught courses in which they had substantial student contact in the classroom (delivering instruction, designing or facilitating activities with students) and agency (e.g., grading, enforcing policies). At the Rocky Mountain institution, there were 16 participating GTAs (exceeding the target enrollment of 15), and at the Southwest institution, there were 27 participating GTAs (exceeding the target enrollment of 20) for a total of 43 GTAs across institutions. Of the 43 GTAs who completed the beginning-of-program survey and 42 who completed the end-of-program survey, 35 completed both; eight completed only the beginning survey and seven completed only the end survey. The discrepancy in numbers is because some GTAs attended an informational meeting and completed the pre-survey but could not attend all sessions, and some GTAs who participated did not complete the pre-survey. Not all students' beginning- and end-of-program survey responses could be linked at the individual level. Lead GTAs were included in the sample and were not separated out, given the small sample size. Of the 43 participants who completed the surveys, 42 responded to the race and ethnicity question: 19 (45%) identified as a race or ethnicity other than white, 13 (31%) identified as white, and 10 (24%) chose not to disclose their racial or ethnic identity. There was a lower proportion of students of underrepresented ethnic and racial minorities among GTA participants compared to the undergraduate populations of the two institutions. Of the 42 GTAs who reported their degree program, 23 (55%) were PhD students, 18 (43%) were MS/MA students, and 1 (2%) selected “other”; 16 (38%) were in their first year of their program, 8 (19%) in their second year, 7 (17%) in their third year, 5 (12%) in their fourth year, 4 (10%) in their fifth year, and 2 (5%) in their sixth year or beyond.

### Survey instrument and measures

GTAs at both institutions completed surveys at the beginning and end of the program to evaluate the impact of the SEP-GTA Program. Surveys were developed by one of the organizational partners and were aligned to the constructs of the GTA training. Surveys were administered in Qualtrics, once at the beginning of the program, and once after program completion. Data were reviewed and analyzed by one of the organizational partners, and shared with workshop leaders at the end of the semester.

The survey included Likert-scale items related to GTA beliefs and perceptions related to mindset, teaching self-efficacy, evidence-based teaching practices (ETPs), and departmental support for teaching, as well as self-reported usage of ETPs (Supplemental Table S2). The survey included 32 items across four constructs (four items measuring mindset beliefs, two items measuring teaching self-efficacy, four items measuring GTA training support, six items measuring perceived feasibility and cost of ETPs), and 16 items assessing self-reported ETP usage. In the context of the survey, we define ETPs as specific actions or strategies that GTAs could use to enact one or more of the targets of the training (e.g., promoting growth mindset, belonging). The survey also included demographic and financial security questions, which were excluded from the analysis because of small sample size and risk of identifiability. Both surveys included one open-ended question. The beginning-of-program survey asked GTAs to describe any concerns about using evidence-based teaching practices; this question was included to inform program facilitators ahead of the training. The end-of-program survey asked GTAs to describe the impact of the program on themselves and their students, which was used to contextualize and add validity to inferences from the quantitative data. Survey items for both surveys were adapted from previous projects on the applied use of ETPs, including the SEP (Student Experience Project 2022). The 16 ETPs included in the survey were selected from a previously developed set of practices, vetted with faculty, and refined based on the Lead GTAs’ feedback. The core survey measures have been validated prior to this work with faculty samples across multiple contexts, providing construct, convergent, and predictive validity evidence (see Supplemental Materials Table S2 for full measure citations).

For the multi-item constructs of GTA Mindset Belief and Perceived Department and Instructor Support, we calculated Cronbach’s alpha α to assess internal consistency reliability; values of 0.70 or higher are typically considered acceptable (Cronbach 1951). Cronbach’s alpha is reported only for constructs with two or more items, as reliability coefficients cannot be calculated for single item measures. We combined measure items only if values were over 0.70. The Growth Mindset Composite surrounding intelligence demonstrated good reliability at both the beginning (α = 0.72) and end of the program (α = 0.94), so we grouped those items.

### Summarization and evaluation

To evaluate the impact of program participation on GTA perceptions, beliefs, and behaviors, we examined the frequency of GTAs who fell in our target response category for each question at the end of the program compared to the beginning. We compared aggregate beginning-and-end-of-program frequency of each response category rather than comparing means because Likert scale data are ordinal. We chose not to link individual GTA respondents across time points (beginning- and end-of-program surveys) to maximize sample sizes. We did not separate results by institution because the trends were similar in both direction and magnitude.

## Results of evaluating the SEP-GTA professional development model

### GTA perceptions

In the beginning and end-of-program surveys, GTAs reported their perceptions related to growth mindset and ETPs and about how supported they felt in using ETPs. In descriptive frequency comparisons of beginning-to-end-of program responses, GTAs reported endorsing greater growth mindset beliefs and higher levels of self-efficacy about their abilities to help students learn and grow after their participation in the GTA training program (Table 2). GTAs more frequently reported positive perceptions of their training (e.g., provided appropriate training to learn about and implement the teaching practices) and evidence-based teaching practices (e.g., comfort and familiarity implementing the practices) from beginning to the end of the program. Thus, more GTAs reported endorsing growth mindset beliefs and felt more equipped to implement evidence-based teaching practices at the end of the program. The one exception was perceived instructor of record support, which showed a slight decline from beginning to end of the program (93.0–90.4%), though the majority of GTAs still reported feeling supported by their instructor of record at the end of the program. This slight decline could be because the survey was given at the end of the semester, when GTAs and instructors may be experiencing the strain and fatigue of the semester, so they perceive less support compared to their needs. Responses to the open-ended question on the survey further highlight the value of the GTA training program for supporting GTAs in learning about and implementing ETPs in their courses. Participating GTAs consistently cited growth in their own knowledge and confidence using ETPs, as illustrated in the quotations below:

**Table 2:** Summary of GTA responses to beginning-of-program and end-of-program surveys using frequency analysis

Overarching construct	Construct	Target response	Beginning %	End %	Change
GTA endorsement of growth mindset beliefs	Growth mindset—intelligence	Strongly disagree/disagree	76.7	88.1	+11.4
	Growth mindset—more effort	Strongly disagree/disagree	93.0	97.6	+4.6
	Growth mindset—try easier	Strongly disagree/disagree	58.1	69.0	+10.9
Self efficacy	Teaching self-efficacy—I’m good at helping students	Strongly agree/agree	48.8	78.6	+29.7
	Teaching self-efficacy—my effort may not help all students	Strongly disagree/disagree	34.9	38.1	+3.2
GTA training support for the use of ETPS	Provided with appropriate training to learn about and implement teaching practices	Very much	27.9	59.5	+31.6
	Interest in professional development opportunities	Very much	55.8	73.8	+18.0
	Departmental support	Strongly agree/agree	74.4	85.7	+11.3
	Instructor of record support	Strongly agree/agree	93.0	90.4	-2.5
Perceived feasibility of ETPS	Familiarity with practices	Strongly agree/agree	79.1	95.2	+16.2
	Comfort with practices	Strongly agree/agree	83.7	95.3	+11.6
	ETPs take more time to implement	Strongly disagree/disagree	32.6	33.3	+0.8
	Difficulty of implementation	Strongly disagree/disagree	39.5	47.6	+8.1

*N* = 43 for beginning and *N* = 42 for end. Target responses included responses in the top one or two categories on the Likert scale. For positively worded items, target responses were “Strongly Agree” or “Agree.” For negatively worded items, target responses were “Strongly Disagree” or “Disagree,” as disagreement reflects endorsement of growth mindset beliefs. For GTA training support items, which used a three-point scale (A little bit, Somewhat, Very Much), the target response was the top single category (“Very Much”). The change column reflects the difference in the percentage of GTAs falling in the target response category between the beginning and end of program surveys.

“Participating in this program has significantly impacted both my teaching approach and my students' success. It has provided me with valuable insights into areas for improvement and equipped me with effective strategies to support my students' learning. This experience has been incredibly beneficial, enhancing my understanding and ability to facilitate student success.”

— Southwestern Institution GTA 1

“My students all have significantly different backgrounds and experience in the material and this program gave me the tools necessary to celebrate that diversity and accommodate everyone instead of feeling timid.”

— Southwestern Institution GTA 2

“I feel better equipped to help students grow and learn. I am more confident about myself as a GTA, and that I have resources I need to help my students.”

— Rocky Mountain Institution GTA 1

### ETP usage

GTAs reported how often they used specific teaching practices. As illustrated in Fig. 2, the “Used Before” category reflects self-reported pre-training baseline practice use, and the “Started Due to Program” category reflects new updates attributed to the training. Every practice was used by at least 20% of the GTAs prior to the training. After the training, every practice had an increase in percentage of GTAs using the practice as a result of the training and almost all practices were used by over 80% of GTAs. The practices with the largest increase due to the program (i.e., highest “Started Due to Program” percentages) were: (1) acknowledge to students that it is common to be challenged by course material at some point in the term and that this is an important part of the learning process (61%); (2) explicitly tell students, in any format (e.g., on the syllabus, in announcements, verbally in class), that you believe they are capable of growing their abilities if they put in the time and effort to learn course material and use resources for success (57%); (3) normalize the use of academic resources, reflecting on feedback, and seeking help when needed as learning strategies that are key to success (54%); and (4) communicate to students that you believe they are capable of reaching the standards set for the course (52%). The practice least used before and after training was incorporating the work of scholars from diverse backgrounds (21% used before; 27% hesitant/won’t use). GTAs indicated that they would use this practice in the future. We suggest that this result is reflective of the fact that GTAs in our sample had limited agency over the creation of curriculum for the courses they taught. This pattern is consistent with research on biology educators more broadly: instructors often make reasoned choices based on the resources and constraints of their disciplinary context, not simple resistance to evidence-based practices (Bouwma-Gearhart et al. 2018).

**Fig. 2:**
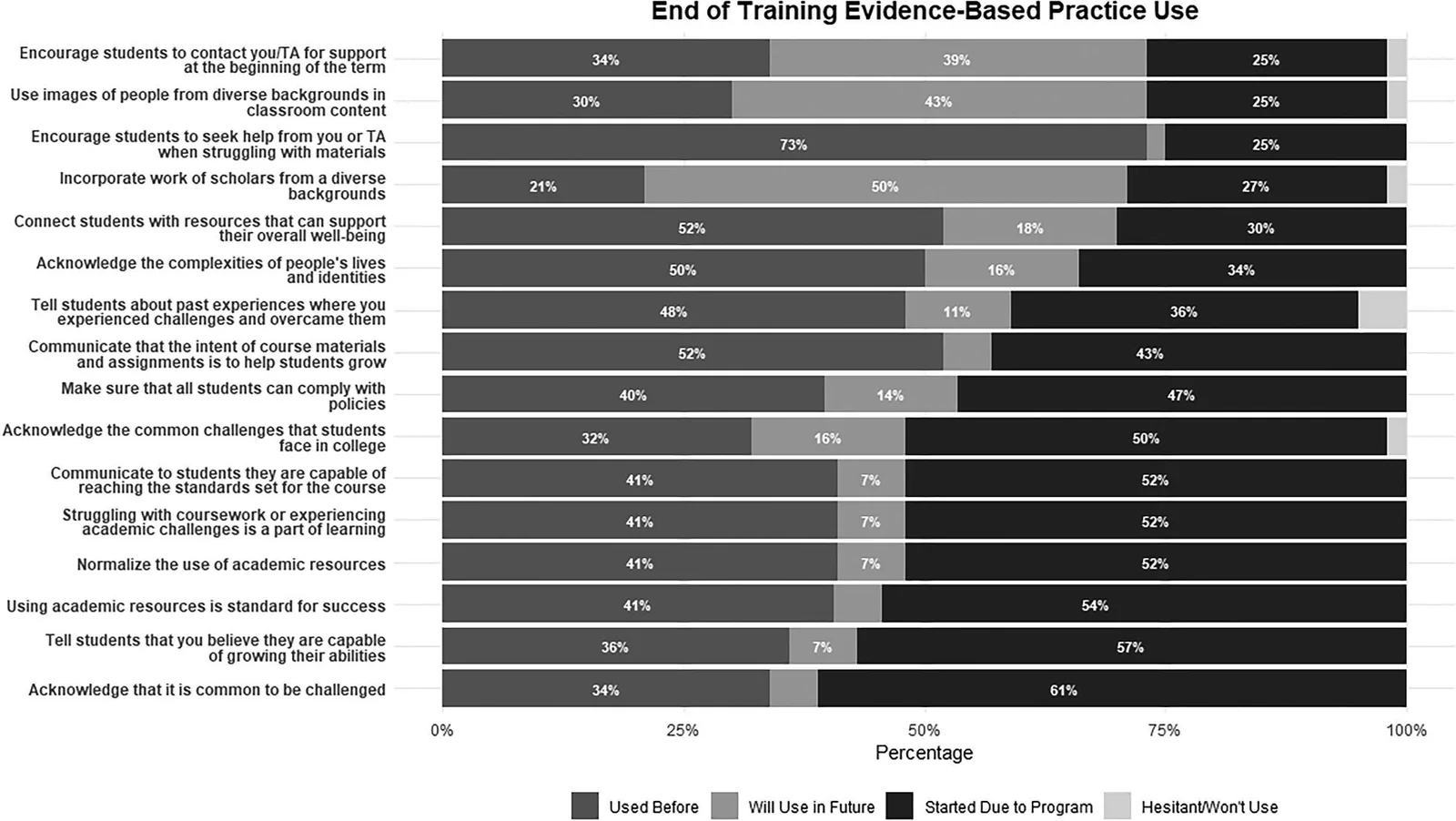
End-of-training evidence-based practice use. Percentage of GTAs on the end-of-program test who already used, planned to use, started to use, or were hesitant to use each specific ETP strategy.

### Downstream impacts on students

While not a goal of this program evaluation, a number of GTAs also reported perceived benefits to their students as a result of implementing ETPs from the GTA training program in their courses.

“I learned several equitable practices and active learning techniques which I was able to implement throughout the semester, and received positive feedback from my students about how those techniques and practices were received or helped them grow and succeed.”

—Rocky Mountain Institution GTA 2

“I was quite happy to read my Faculty Course Questionnaires (Responses) this past year as they mentioned the support aspect as well as meaningful feedback on work.”

—Rocky Mountain Institution GTA 3

“I have received so much positive feedback from my students. I feel that this program has helped me connect with my students more, which has led to higher success for them and a nice confidence boost for myself! After implementing some of the techniques I learned about in this program, I went from ∼5 students attending drop-in hours to ∼50 students attending drop-in!! Also, the class average exam grades went up almost 20%! Thank you!”

— Southwestern Institution GTA 3

“This training has made me more aware of issues that the students are dealing with at home. It has made me more compassionate for their learning and as such has made me more lenient in certain areas of the syllabus. My students also seem happier this semester. One of the classes wants to take a final photo together which has never happened before.”

— Southwestern Institution GTA 4

## Discussion

The SEP faculty and GTA resources are meant to increase use of evidence-based teaching practices and improve academic outcomes (e.g., learning, grade achievement, satisfaction) for all students and are founded on a large body of research on mindset and social belonging psychology. While the SEP resources have been employed at over 50 universities across the country, faculty are typically the target audience. This work illustrates that the SEP resources can be effectively translated to fit the needs of GTAs. The adaptation of the SEP resources to address the unique needs of the GTA context is critical, given the role that GTAs play in STEM education. Our findings demonstrate that the adapted SEP resources produced positive impacts on GTA mindset, perceptions of self-efficacy, perceptions of training support, and knowledge and use of evidence-based teaching practices.

### Implementation similarities and differences between two institutions

Each institution tailored the workshop content, delivery method, and sustainability plans to their needs and institutional context, illustrating that the SEP practice guides resources are adaptable. Each institution selected related readings, videos, or recruited guest speakers (experienced SEP faculty) to help provide background knowledge to GTAs in ways likely to resonate with their population of GTAs and students. Similarly, each institution crafted their own discussion questions, reflection prompts, and activities for GTAs to use to explore their learning. Each institution worked with GTAs to create prompts to gather feedback from their students. Notably, both institutions found distinct ways to sustain the SEP-GTA training at their universities beyond the initial pilot program. The Rocky Mountain Institution incorporated it into an existing course in the Department of Biology and the Southwestern Institution developed a new course in the Department of Biology as well as incorporated the content into an existing interdisciplinary course through the Center for Teaching and Learning. Overall, we found three key factors that emerged and allowed for the successful implementation: flexibility of implementation, a community of practice approach, and buy-in from supervisors and leadership.

The success that we observed across two unique institutional contexts reflects a broader principle that adaptability is crucial for scaling evidence-based interventions across diverse institutional contexts (Walton and Yeager 2020). Research on intervention scalability emphasizes that successful programs must be adapted to local constraints while maintaining fidelity to the core principles (Yeager et al. 2022). Our model also included sense-making of student feedback, partnership among instructors and GTAs, and sustained interactions among GTAs. Shared sensemaking, contextualization, and sustained interaction are attributes of effective change in higher education (Kezar 2018). Based on our implementation at two institutions and prior SEP work with faculty, our findings indicate the SEP framework has these crucial characteristics, maintaining effectiveness across diverse formats and institutional structures. The SEP faculty resources were originally designed to work in a variety of universities across the United States and have been used in departments across STEM and the humanities. Similarly, we found that the resources were easily adapted for use by GTAs in two different university contexts. One caveat is that all GTAs at our pilot institutions had a large amount of student contact in their role. We aren’t sure whether GTAs who have less involved roles (e.g., grading) would benefit as much from the training.

### Community of practice approach

As in the original SEP (Student Experience Project 2022), the CoP approach proved to be important and impactful because it allowed participants to discuss ideas and expand their understanding of the materials. GTAs were able to learn about evidence-based teaching practices, implement them in their classrooms, and then discuss their implementation with their peers shortly after. This collective learning environment facilitated by Lead GTAs created a safe space for GTAs to learn from one another and collaboratively make improvements that considered or incorporated their peers' experiences and feedback. It also provided a chance for Lead GTAs to model the relevant practices (e.g., icebreakers, belonging stories) for participating GTAs.

### Buy-in and support from university leaders

We suggest that buy-in or at least a lack of resistance from GTA supervisors was very important for the successful implementation of the SEP GTA training. The Southwestern Institution had over 10 faculty supervising the GTA participants in their roles, while almost all GTAs at The Rocky Mountain Institution were supervised by two faculty. In our pilot, almost all of the supervisors had previously participated in SEP training or had experience with evidence-based teaching practices. They were informed of the work and supported GTAs in implementing practices and in discussing practices during GTA preparation meetings. Leadership support is crucial for implementing cultural change initiatives (Murphy and Reeves 2019). Within the academic context, faculty supervisors' beliefs about teaching and equity can influence the classroom climate and practices of those who they supervise (Emerson and Murphy 2015). Further, research suggests that when organizational leaders do not support equity-focused initiatives, implementation fidelity suffers and positive outcomes are diminished (Yeager et al. 2022). Finally, teacher change is impacted by tensions with professional identity (Brownell and Tanner 2012; Musgrove et al. 2025). GTAs who are supported in their teaching roles may be less likely to experience tensions with their identity as researchers.

### Our model in context with other GTA training models and research

Our work offers one tractable model for GTA training in inclusive pedagogy. In recent years, there has been an increase in calls for programming and research related to GTA training (e.g., Goodwin et al. 2025; Reeves et al. 2016). GTA training programs include a variety of broad (e.g., student-centered instruction; Pentecost et al. 2012) to more specialized goals (e.g., supporting students in Course Based Undergraduate Research courses; Kern and Olimpo 2023). GTA training to use evidence-based teaching practices has shown benefits for undergraduate student learning, GTA self-efficacy and confidence, greater use of student centered pedagogy, and increased use of inclusive practices (Becker et al. 2017; Batterton et al. 2024; Stromholt et al. 2024). GTA training is often of short duration (i.e., days), but more sustained training that is semester or year long can lead to more durable outcomes (Navlekar et al. 2025).

The model we created is compatible with other models of GTA training and takes into account issues known to influence the impact of GTA training. Our model requires at least one semester of sustained interaction, which is likely to lead to greater impact than a single workshop. It is practice-based in that GTAs learn a teaching practice, implement that practice, and gather and reflect on student-feedback. Our model is unique to our knowledge in that it is grounded in the research base around growth mindset and belonging. We believe our model could be incorporated with training models that address student learning and cognition. Recent research has suggested that impacts of GTA training are influenced by autonomy of GTAs, and that training both impacts and is impacted by GTA self-efficacy (DeChenne et al. 2015; Chiu and Corrigan 2019; Freeman et al. 2025b). As a result of our findings in regards to GTA ETP usage, our model also accounts for GTA limited autonomy by providing practices that can be used in a variety of contexts; it also includes a practice guide designed for GTAs and their supervisors to collaborate on course changes, as a way of increasing GTA agency.

### Implications for STEM equity and student success

Our findings have important implications for addressing equity gaps within biology and more broadly within STEM education. Research consistently demonstrates that students from underrepresented backgrounds face challenges in STEM courses, that is, stereotype threat, belonging uncertainty, and chilly classrooms (Cheryan et al. 2009; Spencer et al. 2016). Inclusive pedagogy training for GTAs who teach foundational STEM courses may allow institutions to create more inclusive learning environments and improve students' success at scale.

The practices that GTAs most frequently implemented after participating in the workshops (acknowledging challenges as normal, expressing belief in student capability, normalizing help seeking) address known barriers to STEM success for underrepresented students. These practices are also examples of what has been termed “Instructor Talk,” language instructors use to build connection and shape classroom climate rather than convey course content (Seidel et al. 2015; Harrison et al. 2019). Research shows that when instructors communicate growth mindset beliefs and normalize students' struggles, students are more likely to persist through academic challenges and maintain their STEM aspirations (Walton et al. 2015; Brady et al. 2020). GTAs’ own reflections on the training further illustrates these shifts, many of the GTAs described feeling newly equipped to support diverse learners and observed positive changes in their students’ engagement and participation. This suggests that the quantitative gains we measured could translate into meaningful classroom experiences for undergraduate students.

### Limitations

Findings from this program evaluation demonstrate promising preliminary evidence for the efficacy of equipping graduate students with evidence-based teaching practices related to mindset and belonging. However, there are several limitations that are worth noting as others consider implementing this model. The sample size across institutions was small, with 43 GTAs across two institutions, meaning findings may not reflect what others would experience. Additionally, although demographic and financial security data were collected, we chose not to report detailed breakdowns given the small sample size, which creates a risk of identification of individual participants. Future implementations with larger samples would benefit from examining whether GTA identity or financial background influences program engagement and outcomes. Both of the institutions are MSIs located in the Mountain West and Southwest, and findings may look different in other institutional contexts. Lead GTAs, who had deeper familiarity with the program and practices, were included in our sample, which may have influenced the results. The GTAs in our sample all had substantial teaching responsibilities, meaning that they had a level of autonomy in the classroom; whether GTAs with more limited roles would have similar outcomes from this training is unknown. A further limitation of our study is that we did not account for GTAs' prior teaching experience, the specific courses they taught, their year in program, or other demographic factors; these variables could all have potentially influenced their survey responses. Lastly, the survey relied on GTA self-reported outcomes and did not include undergraduate student experience or performance data; meaning we were unable to capture whether changes in the GTAs' teaching practices translated to improved undergraduate student success.

## Future directions

By scaling the intervention to more institutions and collecting larger sample sizes among the graduate student trainees and their corresponding undergraduate students, researchers can isolate which evidence-based teaching practices are improved from exposure to the intervention and for which students these practices yield the strongest gains.

Such research could contribute to the growing literature on scalable interventions for improving STEM education equity. A meta-analysis suggests that instructor-focused interventions may be more sustainable and cost-effective than student-focused interventions alone, as they impact multiple student cohorts over time (Walton et al. 2023). Understanding how GTA training translates to student outcomes would inform both theory and emerging practices in the classroom.

Furthermore, by evaluating the intervention across departments with varying degrees of faculty buy-in, researchers can uncover barriers to implementation while identifying possible solutions to increase the scalability and implementation fidelity of the intervention. Future research should also examine longer-term impacts on GTA development and career trajectories, as training in evidence-based teaching practices could impact GTAs' approaches to instruction later as faculty members. These early career interventions could create lasting changes in STEM education culture. Finally, because GTAs are also students, learning about mindset and belonging through this training potentially could impact how GTAs themselves navigate academic struggles and belonging concerns in graduate school. Thus, it is worth exploring whether and how SEP-GTA program participation impacts GTA retention and degree completion.

### Using these resources at your own institution

Even departments actively working to reform undergraduate education can overlook GTA training. Peteroy-Kelly et al. (2019) studied life sciences departments participating in a national recognition program for departmental change and found that GTAs teaching introductory courses in one department received no formal training, and that the department had no way to evaluate their teaching at all. We recommend that universities get started by first assessing their readiness for implementing this type of GTA training to guide planning. The readiness assessment (found at https://studentexperienceproject.org/gta/) includes “defining your why” and “assembling your team.” We also recommend that this work be done in a GTA CoP, as this research-backed approach was found to work for both faculty in the original SEP and GTAs in our study (Vescio et al. 2008). The resources website also provides a GTA toolkit with tailored practice guides that can be used to anchor the agenda and activities for a workshop session, while still allowing universities/departments to flexibly incorporate content suited for their context (e.g., data about student identities at the university). When adapting this model, we encourage institutions to consider a few key factors. First, institutions should consider the level of autonomy GTAs have in their teaching roles alongside the attitudes of their faculty supervisors, as these two factors are intertwined. A GTA who is excited about implementing ETPs but works with a fixed mindset supervisor who is unsupportive or actively opposes the use of ETPs will face significant barriers to implementation. For this reason, securing faculty supervisor buy-in before having GTAs participate in a CoP is something we view as essential, not optional. For institutions where buy-in is not yet fully established, our readiness assessment includes a specific step for this: identifying potential barriers (e.g., faculty skepticism about the value of teaching relative to research) and a plan to address them, such as enlisting a respected faculty or administrative champion who can make the case to colleagues and reinforce that message over time, rather than waiting for full consensus before beginning this work. Institutions where GTAs have limited instructional authority, such as those primarily serving as graders, may find that starting with essential practices and investing in parallel faculty engagement yields better outcomes than GTA training alone. Second, the structure of the CoP can be flexible; it can be embedded into an existing pedagogy course or built into a workshop series. What is the most important is that GTAs have repeated opportunities to learn, implement, and reflect together over time rather than in a standalone workshop. Third, it is also worth considering how GTAs' time will be protected for participation in a CoP. Institutions should think intentionally about how participation will be structured so that it does not feel like an added burden on top of GTAs’ research, teaching, and their academic responsibilities.

## Conclusions

We developed and implemented a model for training GTAs to use evidence-based teaching practices, adapted from the SEP model for faculty. After the training, GTAs had higher endorsement of growth mindset beliefs, increased knowledge and comfort with implementing evidence-based teaching practices, higher interest in teacher professional development, and greater perception of departmental support for using ETPs in the classroom. More than 80% of the GTAs self-reported using evidence-based teaching strategies introduced in the training after participation in the program. The SEP-GTA resources are freely available and designed to be flexible. We hope that institutions ready to invest in their GTAs will find this model as a useful starting point for building more equitable and supportive learning environments where all students feel that they belong and can succeed.

## Supplementary Material

obag052_Supplemental_File

## Data Availability

Due to the sensitive nature of the survey data and conditions of IRB approval protecting participant confidentiality, the data are not available for public access.
