# Supplementary material for: A Professional Development Model for Training Biology GTAs in Equitable Teaching: Implementation and Evaluation at Two Minority-Serving Institutions
Source: Integr Org Biol. 2026 Sep 11;8(1):obag052. doi: 10.1093/iob/obag052 (PMC13650479; doi:10.1093/iob/obag052)
Supplement: Supplementary file 1 [file obag052_supplemental_file.docx]

Supplemental Materials

Supplemental Table S1. Outline of potential session topics, activities, and student feedback prompts for the SEP-GTA Community of Practice. All resource guides are available on the SEP website <https://studentexperienceproject.org/gta/practices/>).

| Practice | Session Description |
| --- | --- |
| Establishing Expectations | Resource Guide: Establishing Expectations, A Growth Mindset Approach Goal: Introduce growth mindset and fixed mindset; provide an overview of the impact of mindset on student success and experiences in educational settings; discuss the importance of normalizing struggle and accessing course resources for your students; discuss campus resources and supports and how to message support to students Potential Activities:   - Read and discuss papers cited in the resource guide. - Co-create or examine a list of course-level and campus level supports for students. - Introduce pertinent history of your institution and/or examine institutional research data about your student body (e.g., first-gen status, commuters, Pell eligibility). - Invite a guest speaker (e.g., faculty expert) to talk about what they do to establish expectations and a growth mindset approach for their students. - Draft messages to give to your students about normalizing struggle in college and using campus resources as part of college experience (highlighting 2 resources).   Potential Feedback Prompts:   - Use a get to know you survey with your students (e.g. what are your goals for taking this course, what concerns do you have, what are you excited about? What do you want me to know about you?) - When you find yourself struggling during the semester, what steps could you take. |
| Wise Feedback Framing Statements | Resource Guide: Crafting a Wise Feedback Framing Statement  Goal: Introduce the research base about why feedback is received differently by different students; introduce the research base about the importance of feedback on student success; introduce wise feedback as an equitable method for giving feedback  Potential Activities:   - Read and discuss papers cited in the resource guide. - Discuss - In what ways do you think some students, when seeking feedback, are going to be received differently based on their identity? How can you take into account social identity threat when giving feedback? - Partner up - Partner 1 explains what the elements of a wise feedback statement are. Partner 2 explains what the potential pitfalls could be and how to avoid them. - Write a paragraph about how wise feedback compares to the feedback practices you know about or have experienced as a teacher and as a learner. Discuss your paragraph with others in a group of 3. - Group up with people teaching the same class as you teach. Come up with a list of 3-5 common mistakes from a recent assignment and discuss what specific actionable feedback you could give to help the students. - Draft a wise feedback framing statement to deliver to students with critical feedback on a key assignment.   Potential Feedback Prompts:   - Based on the feedback you got on your paper, do you feel like you know the next steps you need to do to improve? - How do you feel about the feedback you received? (circle all that apply) demoralized, energized, ready to do the next step, like I’m learning, confused |
| Belonging Stories | Resource Guide: Effective Belonging Messages  Goal: Introduce what belonging and belonging uncertainty are; discuss the research base regarding the importance of belonging to persistence and success in college; introduce belonging stories as an effective practice.  Potential Activities:   - Watch and discuss student stories from the #webelongincollege website. - Read and discuss papers cited in the resource guide. - Reflective Writing - Reflect on a time you felt you didn’t belong in college. Did it get better? What were the steps by you or others that helped? - In a group of 3-4, brainstorm common phrases we hear at universities that may negatively impact belonging (e.g., Look to your left and look to your right, one of you won’t be here at the end.; It’s in the syllabus.; Nobody should have gotten this question wrong). Come up with alternative phrases that promote belonging. - Partner up: Partner 1 explains what the elements of a belonging statement are. Partner 2 explains what the potential pitfalls could be and how to avoid them. - Draft a belonging story and get feedback from a peer. Make an implementation plan for how you will deliver it to students.   Potential Feedback Prompts: Do you feel comfortable in this course? Do you feel like you belong in this course? What are things that contribute to your sense of belonging here? What are things that detract from your sense of belonging here? |
| Encouraging Connections | Resource Guide: Encouraging Connections in the Classroom  Goal: Introduce the research base about the importance of social connections to academic outcomes in college; introduce ways to help students connect with each other and with the instructor.  Potential Activities:   - Read and discuss papers cited in the resource guide. - Draft and practice messages to students that could demystify instructor drop-in hours, - Brainstorm and practice several ice breaker activities that you might use with students. - Have each person read about active learning strategies that can promote student connections and share them (e.g., Think-pair-share, jigsaw, pass the problem, group brainstorming). - Draft and make an implementation plan for an ice breaker activity to use with students. Get feedback from peers. - Draft an active learning strategy for your course that would promote student connections.   Potential Feedback Prompts: If you had a question about this course, what steps could you take to get an answer? Do you feel that you can rely on other students or the instructor in this course for academic support? What helps you feel more connected to students in this class? |
| Syllabus Review | Resource Guide: Syllabus Review  Goal: Introduce the syllabus as a tool for course development; introduce impact of growth vs. fixed mindset messaging in syllabus on students.  Potential Activities:   - Analyze sample syllabi for growth vs. fixed mindset language. - Critically review the syllabus for the course you are teaching with attention to growth vs. fixed mindset messaging and policies. - Collaboratively edit syllabus with faculty supervisor of course.   Potential Feedback Prompt: Take a moment to reflect on your progress this term – what strategies or resources helped you grow the most in this course? How will you apply what you learned this term in your next term? |
| Policy Review | Resource Guide: Course Policy Review  Goal: Review the messages that course policies can convey to students (e.g., growth vs. fixed mindset, social belonging), Discuss student-centered course policies.  Potential Activities:   - Examine course policies for the course you GTA for and brainstorm ways that students with different identities might be affected by those policies. - Collaboratively work with faculty supervisor to modify course policies.   Potential Feedback Prompt: Were there any parts of this course or things I did this term that made you feel positive, understood, and supported in our class? Is there anything you wish I had done differently that would have helped you feel positive, understood, and supported? |

Supplemental Table S2. Graduate Student Teaching Assistant Survey measures. The sample measures included in the beginning-and end-project surveys provided below were adapted from several of EA’s previous projects, including the [Student Experience Project](https://studentexperienceproject.org/wp-content/uploads/Increasing-Equity-in-Student-Experience-Findings-from-a-National-Collaborative.pdf). Measures were adapted from various sources included in citations below.

| Preface: The goal of this survey is to better understand Graduate Student Teaching Assistant (GTA) experiences and comfort using Equitable Teaching Practices in their role as TA’s at [School Name]. Your answers to this survey will help inform the design of the [GTA Program Name].  All responses will remain confidential. No identifiable information will be shared back with your team, or presented in summaries or discussions of the survey results. | | | |
| --- | --- | --- | --- |
| Survey | Construct | Question | Response Options/Scale |
| Beginning  End | GTA’s Mindset Beliefs^1^ | To be honest, students have a certain amount of intelligence, and they really can’t do much to change it. | 1-6: Strongly agree to Strongly disagree |
| Beginning  End | GTA’s Mindset Beliefs^1^ | Your intelligence is something about you that you can’t change very much. | 1-6: Strongly agree to Strongly disagree |
| Beginning  End | Beliefs About the Relationship Between Effort & Ability^2^ | If students are not good at a subject, working hard won’t make them good at it. | 1-6: Strongly agree to Strongly disagree |
| Beginning  End | Beliefs About the Relationship Between Effort & Ability^2^ | If students are not doing well at something, it’s better to try something easier. | 1-6: Strongly agree to Strongly disagree |
| Beginning  End | Teaching Self-efficacy^3^ | I am good at helping all the students in my classes make significant improvement. | 1-6: Strongly agree to Strongly disagree |
| Beginning  End | Teaching Self-efficacy^3^ | Some students are not going to make a lot of progress this year, no matter what I do. | 1-6: Strongly agree to Strongly disagree |
| Beginning  End | Use and Intentions to Use ETPs^4^ | Please let us know whether you have used any of the following teaching practices or strategies as a Graduate Student Teaching Assistant, as well as the likelihood of you using them in the future: [EBT practice] | I already use this strategy (pre)/I used this strategy with students before taking part in this training program;  I started using this strategy with my students because I learned about it in this program (post);  I have not used this strategy, but I would be interested in using it;  I am hesitant to use this strategy with students;  I will not use this strategy with students |
| Equitable teaching practices can be broadly defined as evidence-based teaching strategies or practices that help close opportunity, experience, and outcome gaps between groups by ensuring that all students have access to the resources and support they need to succeed. In this section of the survey, we would like to learn more about your perspective on Equitable Teaching Practices as a GTA. Please indicate how much you agree or disagree with each of the following statements. | | | |
| Beginning  End | Acceptability of ETPs^5^ | I am familiar with Equitable Teaching Practice(s). | 1-6: Strongly agree to Strongly disagree |
| Beginning  End | Perceived Cost of ETPs^6^ | Equitable teaching practices take more time to implement than standard teaching practices. | 1-6: Strongly agree to Strongly disagree |
| Beginning  End | Perceived Cost of ETPs^6^ | Equitable teaching practices are difficult to implement. | 1-6: Strongly agree to Strongly disagree |
| Beginning  End | Perceived Feasibility of ETPs^7^ | My department is supportive of GTA's using equitable teaching practices. | 1-6: Strongly agree to Strongly disagree |
| Beginning  End | Perceived Feasibility of ETPs^7^ | The instructor of record for the class I TA for is supportive of GTA's using equitable teaching practices | 1-6: Strongly agree to Strongly disagree |
| Beginning  End | Perceived Feasibility of ETPs^7^ | I feel comfortable using evidence-based practice(s) as a GTA. | 1-6: Strongly agree to Strongly disagree |
| Beginning  End | GTA Training Support for Use of ETPs^4^ | To what extent do you feel like you are currently provided with appropriate training to learn about and implement teaching practices to make learning environments more equitable for all students? | 1-4: Not at all to very much |
| Beginning  End | GTA Training Support for Use of ETPs^4^ | To what extent would you be interested in having more professional development opportunities to learn about how to quickly and efficiently implement equitable teaching practices? | 1-4: Not at all to very much |
| Beginning | GTA Training Support for Use of ETPs | Do you have any concerns about using equitable teaching practices as a Graduate Student Teaching Assistant? Please explain. | Open-ended |
| End | GTA Training Support for Use of ETPs | What impact, if any, has your participation in this program had on you and your students? | Open-ended |
| Please answer some brief demographic questions. | | | |
| Beginning  End | Gender Identity | With which gender do you identify? | Man  Woman  Non-binary  I identify in another way (please specify):____________  I prefer not to answer |
| Beginning  End | Race/Ethnicity | Which racial/ethnic categories describe you? Please select all that apply. | American Indian or Alaska Native  Asian  Black or African American  Hispanic, Latino, or Spanish Origin  Middle Eastern or North African  Native Hawaiian or Other Pacific Islander  White  Some other race, ethnicity, or origin (please specify):______  I prefer not to answer |
| Beginning  End | What is your current year in graduate school? | What is your current year in graduate school? | 1-6: First year to 6+ years |
| Beginning  End | Program Track | Are you enrolled in a MS/MA program or a PhD program? | MA/MS  PhD |
| Beginning  End | Parent Education | What is the highest level of education completed by either of your parents (or those who raised you)? | Did not finish high school  High school diploma or G.E.D  Attended college but did not complete degree Associate's degree Bachelor's degree  Master's degree  Doctoral or professional degree (PhD., J.D., M.D., etc.)  Other (please specify): |
| Beginning  End | Food Security^8^ | In the past 12 months, was the following situation often true, sometimes true, or never true for you? I worried whether my food would run out before I got money to buy more. | Often True  Sometimes True  Never True |
| In the past 12 months, did you experience any of the following: | | | |
| Beginning  End | Financial security: tuition^9^ | I did not pay my full tuition and fees bill, or I underpaid, because of financial problems | Yes/No |
| Beginning  End | Financial security: housing cost^9^ | I did not pay my rent, mortgage, or housing costs, or I underpaid, because of financial problems | Yes/No |
| Beginning  End | Financial security: housing stability^9^ | I moved in with other people, for any amount of time, because of financial problems | Yes/No |
| Beginning  End | Financial security: homelessness^9^ | I experienced homelessness | Yes/No |

**Survey Measure Citations**

^1^ GTA Mindset Beliefs: These items have been used in prior work to assess college faculty mindset beliefs about student ability (e.g., Canning et al., 2019). In the current study, these items were completed by GTAs in their role as course instructors. These items are derived from highly-validated mindset items developed by Dweck and colleagues (e.g., Dweck, 1999).

Canning EA, Muenks K, Green DJ, Murphy MC. 2019. STEM faculty who believe ability is fixed have larger racial achievement gaps and inspire less student motivation in their classes. Sci Adv. 5(2):eaau4734 <https://doi.org/10.1126/sciadv.aau4734>

Dweck CS. 1999. Self-theories: Their role in motivation, personality, and development. Psychology Press. <https://doi.org/10.4324/9781315783048>

^2^ Beliefs About the Relationship Between Effort & Ability: These items have been used in prior work to assess college faculty beliefs about the relationship between student effort and ability (e.g., Blackwell et al., 2007). In the current study, these items were completed by GTAs in their role as course instructors.

Blackwell LS, Trzesniewski KH, Dweck CS. 2007. Implicit theories of intelligence predict achievement across an adolescent transition: A longitudinal study and an intervention. Child Dev. 78(1):246–263. <https://doi.org/10.1111/j.1467-8624.2007.00995.x>

^3^ Teaching Self-efficacy: These items have been used in prior work to assess K-12 and college faculty teaching self-efficacy beliefs (e.g., Midgley et al., 1989; Miller et al., 2017) and have been extensively validated (e.g., Midgley, 2000). In the current study, these items were completed by GTAs in their role as course instructors.

Midgley C. 2000. Manual for the patterns of adaptive learning scales. University of Michigan. <https://www.researchgate.net/publication/272474856_The_Patterns_of_Adaptive_Learning_Scales_PALS_2000>

Midgley C, Feldlaufer H, Eccles JS. 1989. Change in teacher efficacy and student self- and task-related beliefs in mathematics during the transition to junior high school. J Educ Psychol. 81(2):247–258. [https://doi.org/10.1037/0022-0663.81.2.247](https://psycnet.apa.org/doi/10.1037/0022-0663.81.2.247)

Miller AD, Ramirez EM, Murdock TB. 2017. The influence of teachers' self-efficacy on perceptions: Perceived teacher competence and respect and student effort and achievement. Teach Teach Educ. 64:260–269.<https://doi.org/10.1016/j.tate.2017.02.008>

^4^ Use and Intentions to Use Equitable Teaching Practices, GTA Training Support for Use of Equitable Teaching Practices: These items were created based on implementation fidelity best practices (e.g., Proctor et al., 2011, 2023) and are currently in the process of being validated. Early evidence (Emerson et al., 2023; Ryan et al., 2022) has provided initial evidence of reliability and (construct and predictive) validity.

Emerson K, Hartzog C, Mathias K, Ryan K. 2023. Fostering faculty growth culture practices in California community colleges: Summary report. <https://drive.google.com/file/d/1YPZzopJulnU-I3f4-BIgQjBJKF-U7lPo/view?usp=sharing>

Proctor EK, Bunger AC, Lengnick-Hall R, Gerke DR, Martin JK, Phillips RJ, Swanson JC. 2023. Ten years of implementation outcomes research: A scoping review. Implement Sci. 18:31. <https://doi.org/10.1186/s13012-023-01286-z>

Proctor E, Silmere H, Raghavan R, Hovmand P, Aarons G, Bunger A, Griffey R, Hensley M. 2011. Outcomes for implementation research: Conceptual distinctions, measurement challenges, and research agenda. Adm Policy Ment Health. 38:65–76. <https://doi.org/10.1007/s10488-010-0319-7>

Ryan K, Danek J, Zeribi K, Boucher K, Bartell D. 2022. Increasing equity in college student experience: Findings from a national collaborative. <https://studentexperienceproject.org/wp-content/uploads/Increasing-Equity-in-Student-Experience-Findings-from-a-National-Collaborative.pdf>

^5^ Acceptability of Equitable Teaching Practices: This item was adapted from prior work (e.g., Weiner et al., 2017) to specifically refer to the equity-focused evidence-based teaching practices being provided to GTAs.

Weiner BJ, Lewis CC, Stanick C, Powell BJ, Dorsey CN, Clary AS, Boynton MH, Halko H. 2017. Psychometric assessment of three newly developed implementation outcome measures. Implement Sci. 12:108. <https://doi.org/10.1186/s13012-017-0635-3>

^6^ Perceived Cost of Equitable Teaching Practices: These items were adapted from prior work (e.g., Burden subscale; Aarons et al., 2012) to specifically probe the perceptions and experiences of GTAs (vs. faculty instructors).

Aarons GA, Cafri G, Lugo L, Sawitzky A. 2012. Expanding the domains of attitudes towards evidence-based practice: The evidence based practice attitude scale-50. Adm Policy Ment Health. 39:331–340. <https://doi.org/10.1007/s10488-010-0302-3>

^7^ Perceived Feasibility of Equitable Teaching Practices: These items were adapted from prior work (e.g., EBP Beliefs Scale; Melnyk et al., 2008) to specifically probe the perceptions and experiences of GTAs (vs. faculty instructors).

Melnyk BM, Fineout-Overholt E, Mays MZ. 2008. The evidence-based practice beliefs and implementation scales: Psychometric properties of two new instruments. Worldviews Evid Based Nurs. 5(4):208–216. <https://doi.org/10.1111/j.1741-6787.2008.00126.x>

^8^ Food Security: This item was taken from Center for Community College Student Engagement (2017). Making ends meet: The role of community colleges in student financial health. Austin, TX: The University of Texas at Austin, College of Education, Department of Educational Administration, Program in Higher Education Leadership. <https://cccse.org/sites/default/files/Making_Ends_Meet.pdf>

Center for Community College Student Engagement. 2017. Making ends meet: The role of community colleges in student financial health. University of Texas at Austin, College of Education, Department of Educational Administration, Program in Higher Education Leadership.<https://cccse.org/sites/default/files/Making_Ends_Meet.pdf>

^9^ Financial Security Items: These items were adapted from prior surveys on college student financial insecurity by the [Hope Center for Student Basic Needs](https://hope.temple.edu/research/hope-center-basic-needs-survey/2023-2024-student-basic-needs-survey-report), and are currently in the process of being validated. Early evidence (Ryan et al., 2022) has provided initial evidence of reliability and (construct and predictive) validity.

Hope Center for College, Community, and Justice. 2024. 2023–2024 student basic needs survey report. Temple University.<https://hope.temple.edu/research/hope-center-basic-needs-survey/2023-2024-student-basic-needs-survey-report>

Supplemental Table S3. Courses taught by participating GTAs at each institution during Fall 2023.

| **Institution** | **Courses Taught** |
| --- | --- |
| Southwestern Institution | - Fantastic Fungi - Genes to Genomes - General Microbiology - Human Anatomy & Physiology I and II - Immunology - Medical Microbiology - Molecules to Cells - Organisms to Ecosystems - Principles of Ecology - Technical Writing |
| Rocky Mountain Institution | - Introductory Biology I - Introductory Biology II - Physiology |
